# In-depth characterization of the Wnt-signaling/β-catenin pathway in an in vitro model of Barrett’s sequence

**DOI:** 10.1186/s12876-019-0957-5

**Published:** 2019-03-06

**Authors:** Katharina Götzel, Olga Chemnitzer, Luisa Maurer, Arne Dietrich, Uwe Eichfeld, Orestis Lyros, Yusef Moulla, Stefan Niebisch, Matthias Mehdorn, Boris Jansen-Winkeln, Michael Vieth, Albrecht Hoffmeister, Ines Gockel, René Thieme

**Affiliations:** 1 Department of Visceral, Transplant, Thoracic and Vascular Surgery, University Hospital of Leipzig, Liebigstrasse 20, 04103 Leipzig, Germany; 2 Integrated Research and Treatment Center (IFB) Adiposity Diseases, University Hospital of Leipzig, Philipp-Rosenthal-Strasse 27, 04103 Leipzig, Germany; 3 Institute for Pathology, Klinikum Bayreuth, Preuschwitzer Str. 101, 95445 Bayreuth, Germany; 4 Department of Gastroenterology and Rheumatology, University Hospital of Leipzig, Liebigstrasse 20, 04103 Leipzig, Germany

**Keywords:** Barrett’s esophagus, Esophageal adenocarcinoma, Wnt-signaling, Frizzled receptors, Wnt3a

## Abstract

**Background:**

An altered Wnt-signaling activation has been reported during Barrett’s esophagus progression, but with rarely detected mutations in APC and β-catenin (CTNNB1) genes.

**Methods:**

In this study, a robust in-depth expression pattern analysis of frizzled receptors, co-receptors, the Wnt-ligands Wnt3a and Wnt5a, the Wnt-signaling downstream targets Axin2, and CyclinD1, as well as the activation of the intracellular signaling kinases Akt and GSK3β was performed in an in vitro cell culture model of Barrett’s esophagus. Representing the Barrett’s sequence, we used normal esophageal squamous epithelium (EPC-1, EPC-2), metaplasia (CP-A) and dysplasia (CP-B) to esophageal adenocarcinoma (EAC) cell lines (OE33, OE19) and primary specimens of squamous epithelium, metaplasia and EAC.

**Results:**

A loss of Wnt3a expression was observed beginning from the metaplastic cell line CP-A towards dysplasia (CP-B) and EAC (OE33 and OE19), confirmed by a lower staining index of WNT3A in Barrett’s metaplasia and EAC, than in squamous epithelium specimens. Frizzled 1–10 expression analysis revealed a distinct expression pattern, showing the highest expression for Fzd2, Fzd3, Fzd4, Fzd5, Fzd7, and the co-receptor LRP5/6 in EAC cells, while Fzd3 and Fzd7 were rarely expressed in primary specimens from squamous epithelium.

**Conclusion:**

Despite the absence of an in-depth characterization of Wnt-signaling-associated receptors in Barrett’s esophagus, by showing variations of the Fzd- and co-receptor profiles, we provide evidence to have a significant role during Barrett’s progression and the underlying pathological mechanisms.

**Electronic supplementary material:**

The online version of this article (10.1186/s12876-019-0957-5) contains supplementary material, which is available to authorized users.

## Background

Esophageal cancer is often associated with a poor overall survival when diagnosed in advanced stages [1]. Surgery is the only curative treatment. Available neoadjuvant therapy options prior to oncologic esophagectomy consist of chemotherapy and / or radiochemotherapy [2].

In contrast to the slowly decreasing incidence of squamous cell carcinoma, the incidence of esophageal adenocarcinoma (EAC) has highly increased over the past decades [3, 4].

Mostly, EAC develops from a long history of Barrett’s esophagus, which results from long-lasting gastroesophageal reflux disease (GERD). Barrett’s metaplasia is defined as a condition, where the squamous epithelium of the esophagus is replaced by columnar epithelium with goblet cells [5, 6]. The predominantly benign course of Barrett’s disease is a known risk factor for esophageal adenocarcinoma development [7]. However, an in-depth characterization of the Wnt-receptors along the more advanced stages of this disease is missing.

The Wnt−/β-catenin signaling pathway is responsible for cell growth, motility and differentiation during embryogenesis [8]. In mammals, 19 Wnt molecules are known. In consequence of a non-activated signaling pathway, an intracellular degradation complex composed of glycogen synthase kinase 3 beta (GSK3β), adenomatous polyposis coli (APC) and Axin2 is formed and leads to phosphorylation and ubiquitination of β-catenin [9]. As a result, β-catenin is removed by the proteasomal degradation complex. In activated status, extracellular Wnt molecules bind to membranous frizzled (Fzd) receptors and in combination with Lipoprotein Receptor Related Protein (LRP), they activate the intracellular molecule Dishevelled (Dsh) [10]. Activated Dsh inhibits the formation of the degradation complex. Subsequently, β-catenin accumulates in the cytoplasm and passes to the nucleus, resulting in transcription [8].

In different cancer entities, the Wnt−/β-catenin signaling pathway seems to play a key role during carcinogenesis. In colorectal cancer, a mutation of the APC gene is often observed [11]. In addition, APC gene mutation leads to familiar adenomatous polyposis (FAP), a disease with hundreds of colorectal adenomas with obligatory progression to carcinoma even at a young age [12].

Hepatocellular carcinoma (HCC), hepatoblastoma and hepatocellular adenoma often show mutations of β-catenin [13]. Overall, hepatocellular adenomas with β-catenin mutation have a higher risk of malignant transformation. AXIN1 gene mutation leads to inhibition of the degradation complex with resulting β-catenin accumulation [14].

An activated Wnt−/β-catenin signaling pathway could also be a potential mechanism for the progression of EAC. Clément et al. observed a methylation of the APC promotor, which goes along with a lack of APC expression in Barrett’s esophagus and EAC. In addition to that, a promotor methylation of the Wnt antagonist secreted frizzled receptor protein 1 (SFRP1), which is associated with a loss of function of SFRP1, was found in Barrett’s esophagus and EAC more often than in normal squamous mucosa [15]. Cell culture analysis of the metaplastic cell line CP-A and the esophageal carcinoma cell line OE33 showed higher expression of the Wnt target genes Axin2 and CyclinD1 [16]. Moreover, CyclinD1 and Axin2 were higher expressed in Barrett’s esophagus than in normal squamous epithelium from human specimen biopsies [16]. Furthermore, analysis of β-catenin expression showed high cytoplasmic levels and nuclear accumulation of β-catenin in high-grade dysplasia. A simultaneous reduction of membranous β-catenin expression was found as well [17].

Overall, these findings strongly indicate an activation of the Wnt−/β-catenin signaling pathway during EAC development. In this study, we aimed to investigate the role of Wnt−/β-catenin signaling pathway activation in different stages during the progression from squamous epithelium to EAC in vitro. Analyzing the expression of Wnt3a, activating the canonical, and Wnt5a, the non-canonical Wnt−/β-catenin signaling pathway, membranous Fzd-receptors, intracellular molecules and downstream targets, we want to give a brief overview of Wnt-pathway genes and a comprehensive understanding of the molecular background for Wnt-signaling in EAC progression in an in vitro Barrett’s cell culture model. Nevertheless, we provide exemplary insights in the presence of Wnt-molecules and -receptors in specimens from patients with Barrett’s esophagus, high-grade intraepithelial neoplasia, and esophageal adenocarcinoma.

## Methods

### Human biopsy specimens

For expression analysis of the Wnt-pathway components, endoscopic biopsies were taken from Barrett’s mucosa with intestinal metaplasia, high-grade intraepithelial neoplasia, esophageal adenocarcinoma. Normal mucosa was taken from patients with Barrett’s mucosa using a safety distance of at least 3 cm to the Barrett’s mucosa. Biopsies were placed directly to RNA*later*® (Sigma-Aldrich, Taufkirchen, Germany) for a maximum of 24 h and stored at - 80 °C until further usage. Seven specimens of squamous epithelium, Barrett’s mucosa, HGIN, and EAC were investigated. The diagnoses of Barrett’s mucosa, HGIN, or EAC were confirmed by an expertized GI pathologist.

### Reagents and antibodies

Recombinant human Wnt3a (rhWnt3a, 200 ng/mL) was purchased from R&D Systems (Minneapolis, USA). The following antibodies phospho-GSK3β-Ser9 (D85E12), GSK3β (D5C5Z), phospho-Akt-Ser473 (D9E), Akt (C67E7), phospho-β-catenin-Ser552 (D8E11), and β-catenin (D10A8) were all purchased from Cell Signaling Technology (Danvers, USA) and the β-actin antibody from Sigma-Aldrich (Taufkirchen, Germany). The peroxidase-conjugated goat anti-rabbit and goat anti-mouse antibodies were purchased from Jackson ImmunoResearch (Suffolk, UK).

### Cell culture

In total, six different cell lines, representing Barrett’s sequence, were cultivated. EPC1-hTERT (EPC-1) and EPC2-hTERT (EPC-2) were a generous gift from Dr. Hiroshi Nakagawa [18]. They are immortalized human squamous esophageal cells and were cultivated as previously described [19]. The metaplastic cell line CP-A (CRL-4027) and the dysplastic cell line CP-B (CRL-4028) were purchased from LGC Standards (Wesel, Germany) and cultivated in MCDB 153 growth medium (Biochrom, Berlin, Germany) with supplements according to the manufacturer’s protocol. Originating from esophageal adenocarcinomas, OE33 (ECACC-96070808) and OE19 (ECACC-96071721), cells were acquired from Sigma-Aldrich (Taufkirchen, Germany). They were cultivated in RPMI 1640 growth medium (Gibco, Waltham, USA), supplemented with 10% FBS (Biochrom, Berlin, Germany). All cell lines were incubated at 37 °C with 5% CO_2_.

### Wnt3a treatment

EPC-1, EPC-2, CP-A, CP-B, OE33 and OE19 cells with a confluence of about 80% were seeded with 250,000 cells per well in a 6-well plate and incubated at 37 °C with 5% CO_2_. After one day, the medium was changed and one day later, cells were incubated with FCS free medium for three hours. The cells were stimulated with 200 ng rhWnt3a at 37 °C, 5% CO_2_ for one hour. Removing medium and washing two times with cold PBS, the stimulation with Wnt3a was stopped.

### RNA isolation and cDNA synthesis

For RNA isolation from cell cultures, the RNeasy Plus Mini Kit (Qiagen, Hilden, Germany) was used. RNA isolation from biopsies was done with TRI Reagent® (Sigma-Aldrich, Taufkirchen, Germany), followed by DNase (NEB, Frankfurt a.M., Germany) treatment. CDNAs were synthesized from 250 ng to 1000 ng of total RNA using the RevertAid RT synthesis kit (Thermo Scientific, Darmstadt, Germany) according to the manufacturer’s protocol.

### RNA isolation from FFPE specimens

For isolating RNA from paraffin embedded biopsies, we used the AllPrep DNA/RNA FFPE Kit (Qiagen, Hilden, Germany) according to the manufacturer’s protocol. 125 ng of total RNA were used to synthesize cDNA using SuperScript IV Reverse Transcriptase (Invitrogen, Darmstadt, Germany).

### Quantitative RT-PCR

Quantitative RT-PCR was conducted by using Takyon No ROX SYBR®Core Kit dTTP Blue (Eurogentec, Lüttich, Belgium) with 1 μl of cDNA and 14 μl of reaction mixture. Primers used for quantitative RT PCR are shown in Table 1. Expression of Wnt3a was analyzed using a TaqMan Assay (Hs00263977_m1, ThermoFisher, Darmstadt, Germany) in combination with Blue Probe qPCR Kit (Biozym, Hessisch Oldendorf, Germany). Normalization was done for β-actin expression.

**Table 1:** Oligo sequences

Name	Forward	Reverse	Reference	Length (bp)
Wnt3a	ATGAGCGTGTCACTGCAAAG	TGTTGGGCCACAGTATTCCT	NM_033131	302
Wnt5a	AGAAGAAACTGTGCCACTTGTATCAG	CCTTCGATGTCGGAATTGATACT	NM_003392	101
Fzd1	ATCGAAGCCAACTCACAGTATTT	CACGTTGTTAAGCCCCACG	NM_003505	135
Fzd2	GACCAGGTGAGGATCCAGAG	AGCTACAAGTTTCTGGGCGA	NM_001466	122
Fzd3	TCAAGTCTGGACGACTCATTTG	GCATCTGGGAAACAACGTG	NM_017412	93
Fzd4	TCTTCTCTGTGCACATTGGC	GACAACTTTCACACCGCTCA	NM_012193	92
Fzd5	GAGAGACGGTTAGGGCTCG	GTGACCCAGGGACGGAG	NM_003468	104
Fzd6	ATTCCAGATTTGCGAGAGGA	AAAATGGCCTACAACATGACG	NM_003506	106
Fzd7	GTCGTGTTTCATGATGGTGC	CGCCTCTGTTCGTCTACCTC	NM_003507	92
Fzd8	GACACGAAGAGGTAGCAGGC	CACCGTCTCCACCTTCCTTA	NM_031866	90
Fzd9	AAGTCCATGTTGAGGCGTTC	GAAGCTGGAGAAGCTCATGG	NM_0035.8	108
Fzd10	CAACCAAGAAAAGCACCACA	TATGAGATCCCTGCCCAGTC	NM_007197	98
LRP5	GAGATCCTCCGTAGGTCCGT	CCAAGCGAGCCTTTCTACAC	NM_002335	128
LRP6	AGCGACTTGAACCATCCATT	GAGTTGGATCAACCCAGAGC	NM_002336	115
Axin2	GCAGATCCGAGAGGATGAAG	GGAGTGGTACTGCGAATGGT	NM_004655	250
CyclinD1	GGCGGATTGGAAATGAACTT	TCCTCTCCAAAATGCCAGAG	NM_053056	109
β-Actin	CACTCTTCCAGCCTTCCTTC	GGTGTAACGCAACTAAGTCATAG	NM_001101.3	378

### Qualitative RT- PCR

Using DreamTaq Green PCR Master Mix (Thermo Scientific, Darmstadt, Germany), polymerase chain reaction was performed with 1 μl of cDNA per 20 μl reaction mix. The following protocol was used for 40 cycles: denaturation 98 °C for 2 min, annealing 94 °C for 10 s, elongation 60 °C for 20 s. 10 μl of cycled PCR products were applied to 2% ethidiumbromid stained agarose gel and visualized with UV light. Primers used for qualitative RT PCR are shown in Table 1.

### Westernblot analysis

Harvested cells were lysed in RIPA buffer. Using the method of Bradford, protein concentrations were detected. 20 μg of protein were separated on 8% or 10% sodium dodecyl sulphate (SDS)-polyacrylamide gels and blotted on nitrocellulose membranes. After blocking the membranes with 5% low fat milk for one hour, they were incubated with a specific primary antibody over night at 4 °C. Detecting bound antibody, a peroxidase conjugated secondary antibody goat anti-mouse or goat anti-rabbit was used incubating for 1 h at room temperature. Protein bands were visualized with ECL chemiluminescence detection (Millipore, Billerica, USA). The dilutions of the different antibodies are shown in Table 2. Westernblots were analyzed densitometrically using ImageJ.

**Table 2:** Antibodies

	Dilution	Company	Catalogue number
Primary antibodies			
pGSK3β-Ser9	1:1000 (1% BSA/PBS)	Cell Signalling Technology, Danvers, USA	5558
pβ-catenin-Ser552	1:1000 (1% BSA/PBS)	Cell Signalling Technology, Danvers, USA	5651
pAkt-Ser473	1:1000 (1% BSA/PBS)	Cell Signalling Technology, Danvers, USA	4060
GSK3β	1:1000 (1% BSA/PBS)	Cell Signalling Technology, Danvers, USA	12456
β-catenin	1:1000 (1% BSA/PBS)	Cell Signalling Technology, Danvers, USA	8480
Akt	1:1000 (1% BSA/PBS)	Cell Signalling Technology, Danvers, USA	4691
β-Actin	1:2500 (0.5% LFM/PBS)	Sigma-Aldrich, Taufkirchen, Germany	A1978
Secondary antibodies			
Goat anti-rabbit	1:7500 (0.5% LFM)	Jackson Immuno Research, Suffolk, UK	111–035-045
Goat anti-mouse	1:7500 (0.5% LFM).	Jackson Immuno Research, Suffolk, UK	115–035-068

BSA: bovine serum albumin; LFM: low fat milk; PBS: phosphate buffered saline

### Immunohistochemistry

Paraffin embedded biopsies from squamous epithelium, Barrett’s metaplasia, HGIN and EAC were cut in 3 μm sections and deparaffinized using a series of different concentrated alcohols and xylol. Endogenous peroxidase was blocked by 3% H_2_O_2_ for 20 min at 4 °C. The slices were cooked in citrate buffer with a pressure cooker for 10 min. Blocking unspecific bindings was performed in several steps, first with 5% NormalGoatSerum (Jackson Immuno Research, Suffolk, UK) for 20 min at room temperature and second with Reagents from the Streptavidin/Biotin blocking kit (GeneTex, Irvine, CA), as described in the manufacturer’s protocol. Diluted 1:50 in 1% BSA in PBS, the monoclonal primary antibody against WNT3A (AM09053PU-N, OriGene Technologies GmbH, Herford, Germany) was incubated over night at 4 °C. Detecting bound antibody, a biotin-SP conjugated goat anti-mouse (Jackson Immuno Research, Suffolk, UK) and a peroxidase conjugated Streptavidin (Jackson Immuno Research, Suffolk, UK), both diluted 1:1000 in 1% BSA in PBS, were utilized for 1 h at room temperature. Results were visualized with diaminobenzidine and counterstained with hematoxylin. Breast cancer tissue was used as positive control. Evaluation was done using a staining index (1 < 30%, 2 < 60%, 3 < 100% stained) with categorization for analyzing the stroma, the squamous epithelium, and the metaplastic or EAC tissue.

### Statistical analysis

Analyses were performed using GraphPad Prism 6 (GraphPad Software Inc., San Diego, USA). Results are presented as mean ± standard error (S.E.M.) and a *p*-value of less than 0.05 was considered statistically significant.

## Results

### Expression of extracellular ligands Wnt5a and Wnt3a

**Fig. 1:**
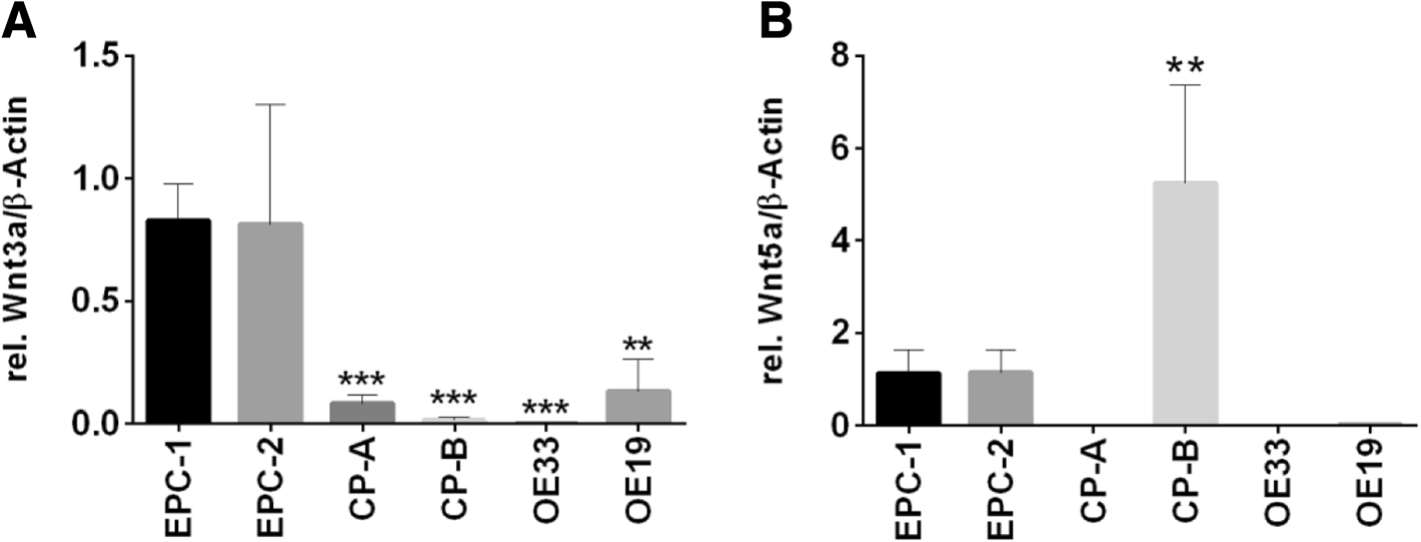
Expression of the extracellular ligands Wnt3a and Wnt5a along Barrett’s sequence. Expression of Wnt3a (**a**) and Wnt5a (**b**) was analyzed in EPC-1 and EPC-2 (squamous esophageal epithelium), CP-A (metaplastic epithelium), CP-B (epithelium with high-grade dysplasia), OE33 and OE19 (esophageal adenocarcinoma) cells by quantitative Real time RT-PCR. Highest Wnt3a expression was detected in EPC-1 and EPC-2 (**a**). Wnt5a was also expressed in EPC-1 and EPC-2, but highest levels of Wnt5a were found in CP-B (**b**). OE33 and OE19 expressed only marginal levels of Wnt3a and Wnt5a (**a**, **b**). Normalization was done with β-Actin. Values are shown as mean ± S.E.M. (One-way-ANOVA with Bonferroni correction, * - *p* < 0.05, ** - *p* < 0.01, *** - *p* < 0.001 compared to EPC-1)

Along Barrett’s sequence, Wnt3a and Wnt5a were expressed differently. Significantly elevated Wnt3a expression levels were detected in squamous cell lines EPC-1 and EPC-2, while metaplastic (CP-A), dysplastic (CP-B) and carcinoma cell lines (OE33, OE19) showed only marginal expression of Wnt3a. Wnt5a was expressed in EPC-1 and EPC-2. However, the dysplastic cell line CP-B demonstrated the highest expression levels of Wnt5a. In contrast, the metaplastic cell line CP-A and the carcinoma cell lines OE33 and OE19 did not express Wnt5a (Fig. 1).

### Expression pattern of membranous Fzd receptors and co-receptors LRP5/LRP6

**Fig. 2:**
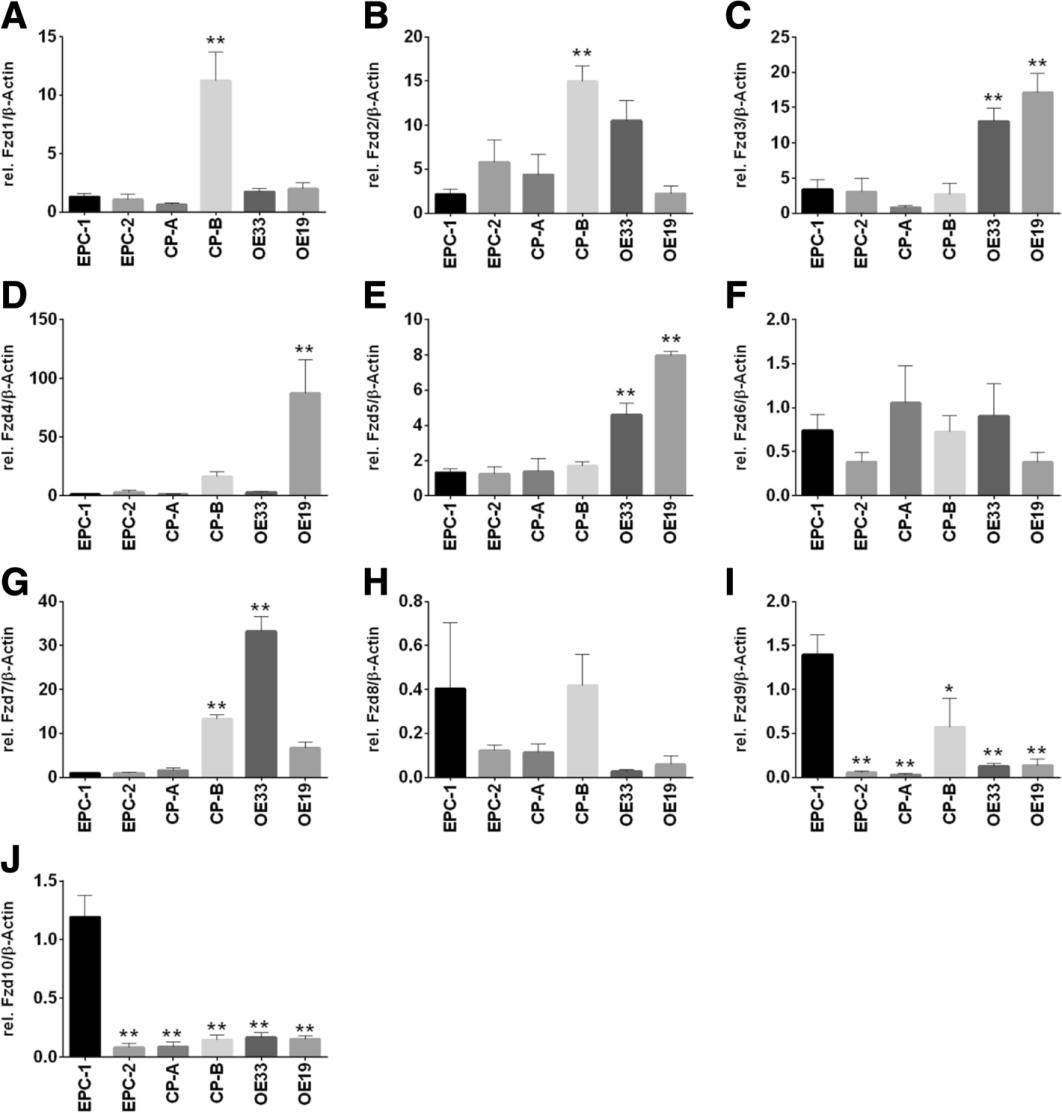
Expression of the membranous receptors Fzd1–10 along Barrett’s sequence. Expression of the receptors Fzd1–10 (**a**-**j**) were analyzed in EPC-1 and EPC-2, CP-A, CP-B, OE33 and OE19 cells by quantitative Real time RT-PCR. Highest expression levels of Fzd1 (**a**) and Fzd2 (**b**) were detected in CP-B. The receptors Fzd3 (**c**), Fzd4 (**d**) and Fzd5 (**e**) were strongly expressed in OE19. Overall, the expression of Fzd6 (**f**) and Fzd8 (**h**) were marginal and varied during Barrett’s sequence. The highest expression of Fzd7 (**g**) was found in OE33. The epithelial cell line EPC-1 expressed the highest levels of Fzd9 (**i**) and Fzd10 (**j**). Normalization was done with β-Actin. Values are shown as mean ± S.E.M. (One-way-ANOVA with Bonferroni correction, * - *p* < 0.05, **- *p* < 0.01 compared to EPC-1)

We found an aberrant expression pattern of the membranous receptors Fzd 1–10 in the investigated cell lines of the Barrett’s sequence model. An increase of Fzd1–5 and Fzd7 expression levels was detected in all cell lines along Barrett’s sequence, especially in CP-B, OE33 and OE19. In contrast, Fzd6, Fzd8, Fzd9, and Fzd10 were only expressed in marginal levels in all cell lines with elevated expressions in EPC-1 (Fig. 2).

**Fig. 3:**
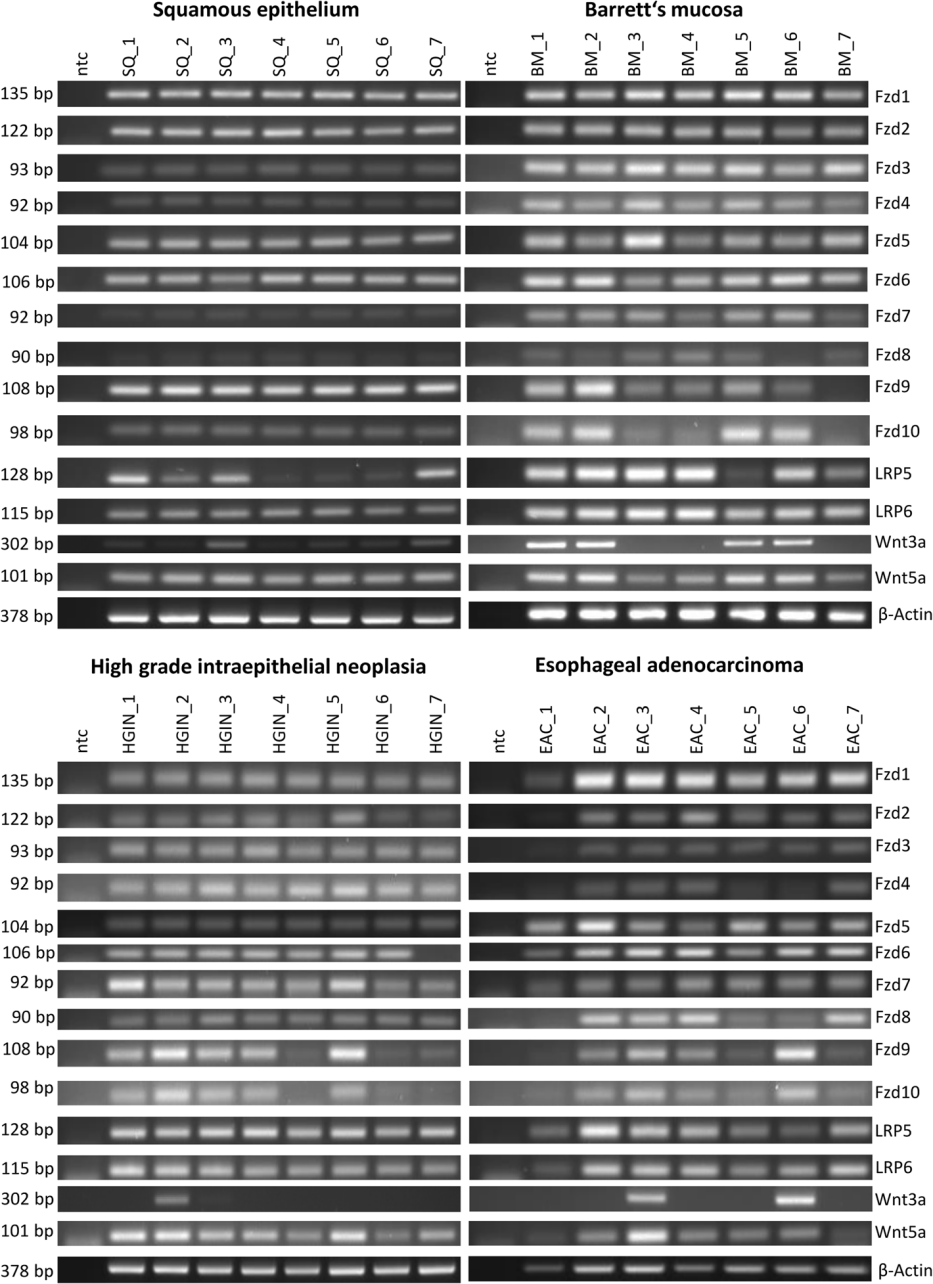
Expression pattern of different Wnt-signaling molecules in patients’ biopsies from squamous epithelium, Barrett’s mucosa, HGIN and EAC. Expression of the receptors Fzd1–10, the co-receptors LRP5/6 and ligands Wnt3a and Wnt5a were analyzed by RT-PCR in biopsies taken during endoscopy from squamous epithelium (SQ_1–7) and their corresponding Barrett’s mucosa (BM_1–7), as well as in specimens from patients with high-grade intraepithelial neoplasia (HGIN_1–7) and esophageal adenocarcinoma (EAC_1–7). Biopsies were placed directly in RNAlater®. PCR products were applied to 2% ethidiumbromid stained agarose and visualized with UV light

To investigate the frizzled receptor expression in humans, we used seven specimens from squamous epithelium, which displayed a high expression of Fzd1, 2, 5, 6, 9, and 10. Fzd3, 4, 7, and 8 showed a distinct or weak expression pattern in specimens from squamous epithelium. Seven Specimens of Barrett’s mucosa, HGIN and EAC showed a homogenous expression of Frz1–7 compared to squamous epithelium. Fzd8 was less expressed in squamous epithelium than in Barrett’s mucosa, HGIN and EAC. While Fzd9 and Fzd10 had a homogeneous expression pattern in the specimens of squamous epithelium, the expression intensity was heterogeneous in Barrett’s mucosa, HGIN and EAC specimens. The Wnt-signaling pathway co-receptor LRP5 displayed a heterogeneous expression pattern in squamous epithelium, while the expression pattern in specimens of Barrett’s mucosa, Barrett’s mucosa, HGIN and EAC were more robust and intensive. LRP6 showed a moderate homogeneous expression in all specimens investigated. As in the epithelial cells EPC-1 and EPC-2, samples from squamous epithelium displayed a weak mRNA-expression of Wnt3a (6 of 7) and a moderate expression of Wnt5a. However, 4 of 7 specimens of Barrett’s mucosa, 1 of 7 specimens of HGIN, and 2 of 7 specimens from EAC displayed a Wnt3a expression (Fig. 3).

**Fig. 4:**
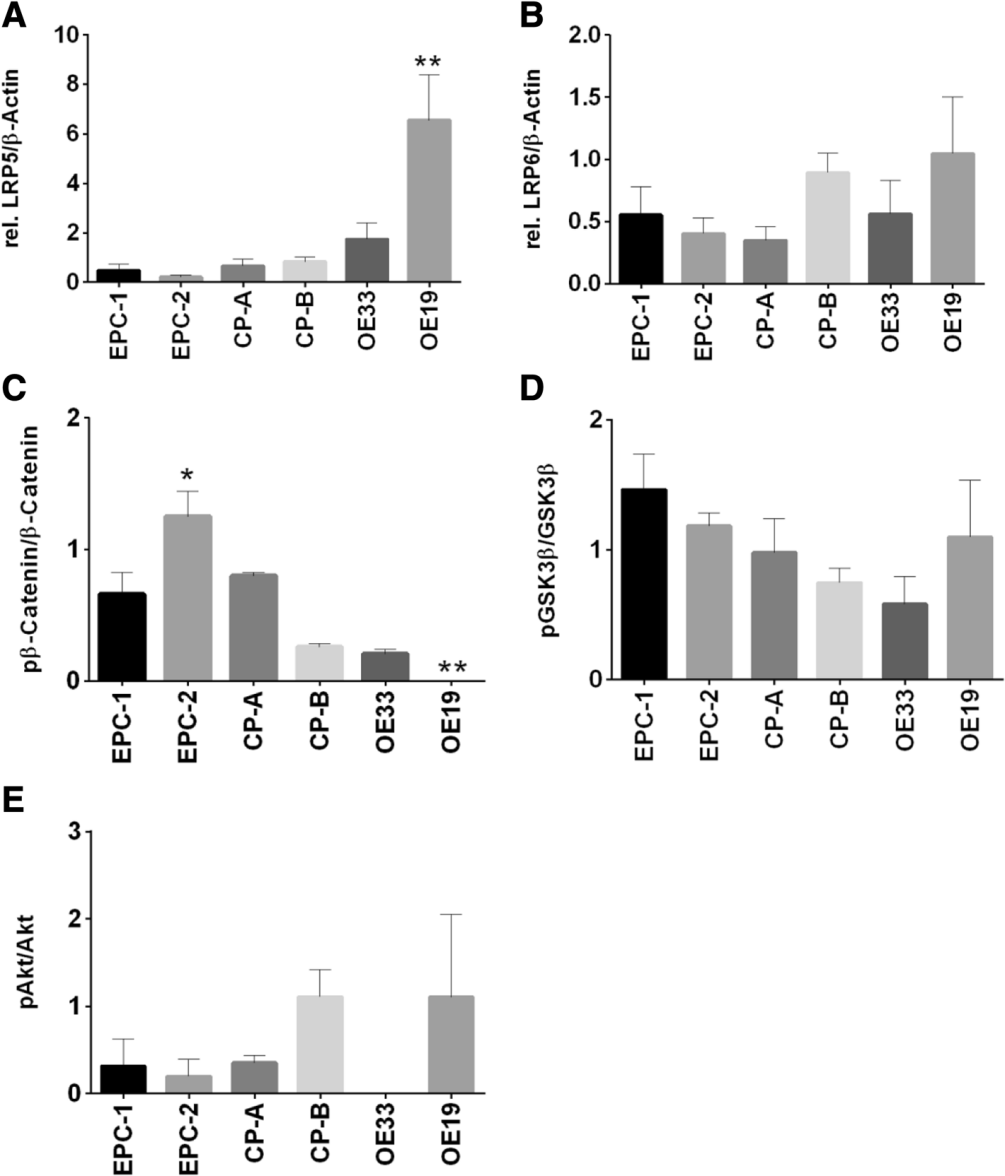
Expression of the co-receptors LRP5/6 and protein levels of β-catenin, GSK3β and Akt. Expression of LRP5 and LRP6 was analyzed in EPC-1 and EPC-2, CP-A, CP-B, OE33 and OE19 cells by quantitative Real time RT-PCR. Along Barrett’s sequence, an increase of LRP5 (**a**) and LRP6 (**b**) levels was detected with highest expression levels in OE19. Protein level expression of pAkt/Akt, pβ-catenin/β-catenin and pGSK3β/GSK3β was analyzed in EPC-1 and EPC-2, CP-A, CP-B, OE33 and OE19 cells by westernblot analysis. A significant higher pβ-catenin expression was found in EPC-2. (**c**). Elevated levels of pGSK3β were detected in EPC-1, EPC-2, and OE19 (**d**). EPC-1, EPC-2 and CP-A expressed only marginal levels of pAkt. A loss of pAkt expression was detected in OE33 (**e**). Normalization was done with β-Actin. Values are shown as mean ± S.E.M. (One-way-ANOVA with Bonferroni correction, * - *p* < 0.05, **- p < 0.01 compared to EPC-1)

Additionally, we found increasing expression levels of the co-receptors LRP5 and LRP6 along Barrett’s sequence. Lowest expression levels were detected in the epithelial cell line EPC-1, and highest levels in the carcinoma cell line OE19 (Fig. 4a-b).

### Activation of ß-catenin, GSK3ß and Akt in Barrett’s esophagus

Westernblot analyses of phosphorylated β-catenin (Ser552) displayed differences between the squamous cell lines EPC-1 and EPC-2, with significant higher levels of pβ-catenin in EPC-2. Overall, EPC-1, CP-A, CP-B, and OE 33 showed low levels of pβ-catenin. The presence of pβ-catenin could not be detected in OE19.

We revealed elevated expression levels of pGSK3β (Ser9) in the squamous cell lines EPC-1 and EPC-2, as well as in the carcinoma cell line OE19. In contrast, OE33 showed a low expression of pGSK3β. The metaplastic cell line CP-A expressed balanced levels of pGSK3β and GSK3β. Analysis of the kinase Akt showed different protein expression levels of phosphorylated Akt (Ser473) in the different cell lines. The cell lines EPC-1, EPC-2 and CP-A expressed only marginal levels of pAkt. Balanced expression of pAkt and Akt was found in CP-B and OE19. We detected a loss of pAkt expression in the carcinoma cell line OE33 (Fig. 4c-e).

### Expression of the downstream target Axin2

**Fig. 5:**
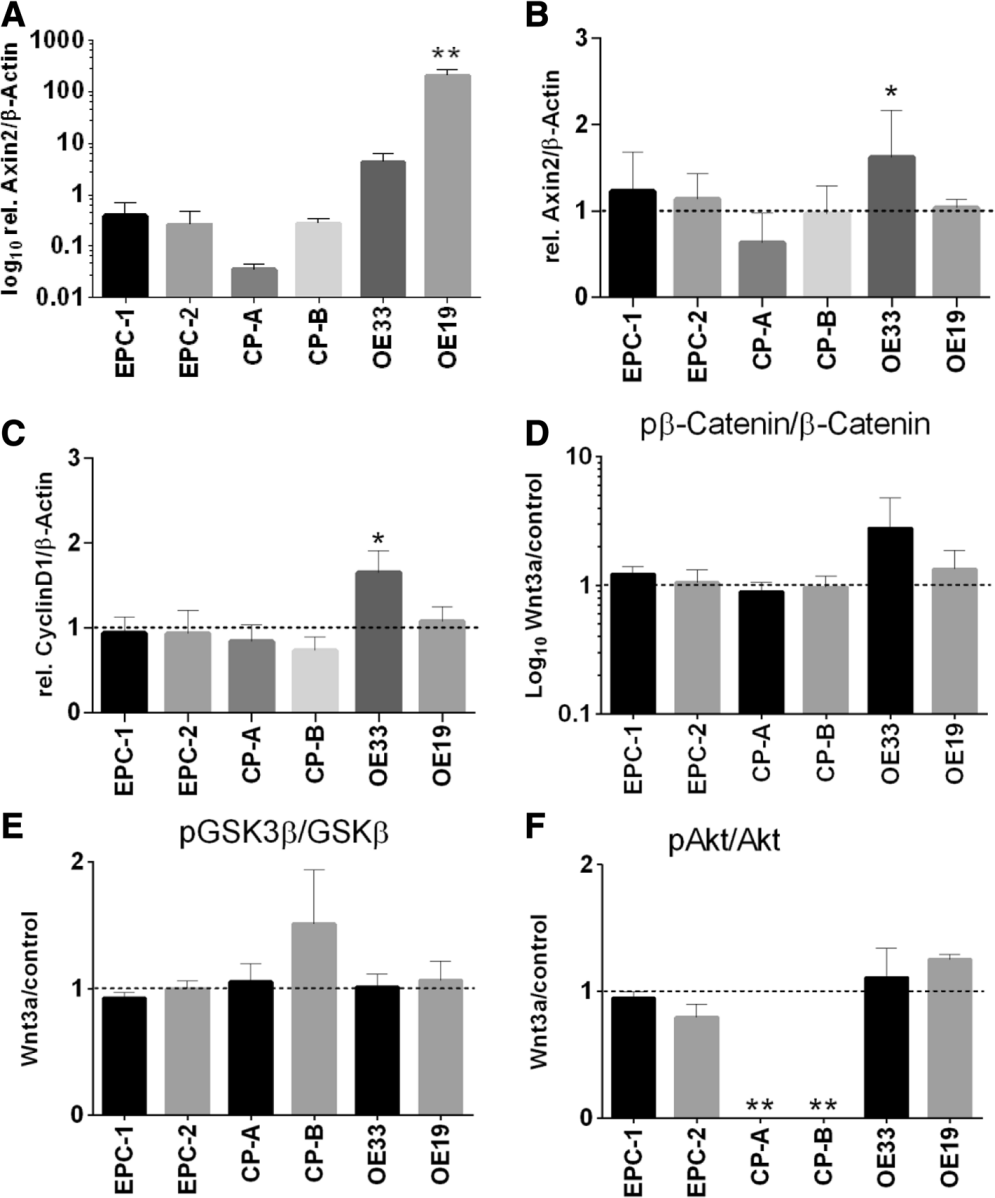
Response to Wnt3a in cells of Barrett’s sequence. Expression of the downstream target Axin2 was analyzed in EPC-1 and EPC-2, CP-A, CP-B, OE33 and OE19 cells by quantitative Real time RT-PCR (**a**). The carcinoma cell lines OE33 and OE19 were treated with 200 ng/mL rhWnt3a for 1 h. Cell specific FCS free medium served as control. Expression of the downstream targets Axin2 and CyclinD1 were analyzed by quantitative Real time RT-PCR (**b**, **c**). Along Barrett’s sequence, a strong increase of Axin2 expression was found with significant higher levels in OE19 as compared to EPC-1 (**a**). EPC-1, CP-A and OE33 cells responded to Wnt3a with an increased Axin2 expression. In contrast, EPC-2 and OE19 showed no altered Axin2 expression as compared to controls (**b**). Decreased levels of CyclinD1 after Wnt3a were detected in EPC-1, EPC-2, CP-A, and CP-B. Wnt3a treatment induced significant higher CyclinD1 levels in OE33 cells as compared to controls (**c**). Protein level expression of pAkt/Akt, pβ-catenin/β-catenin and pGSK3β/GSK3β after Wnt3a treatment was analyzed in EPC-1 and EPC-2, CP-A, CP-B, OE33 and OE19 cells by westernblot analyses. Higher levels of pβ-catenin after Wnt3a treatment were found in OE33 cells (**d**). CP-B responded with an elevated pGSK3β expression (**e**). A loss of pAkt was detected in CP-A and CP-B (**f**). Normalization was done with β-Actin. Values are shown as mean ± S.E.M. (One-way-ANOVA with Bonferroni correction, **- p < 0.01 compared to EPC-1 (**a**), Two-way-ANOVA with Bonferroni correction, * - *p* < 0.05, ** - *p* < 0.01 compared to controls (**b**-**f**))

Expression of the downstream target Axin2 increased significantly along Barrett’s sequence, with 516 times higher expression levels in OE19 than in EPC-1 cells (Fig. 5a).

### Response to Wnt3a in cells of the Barrett’s sequence

Cells from all stages of Barrett’s sequence were treated with Wnt3a to determine differences in Wnt3a receptiveness. After Wnt3a treatment (200 ng/mL) for 1 h, we analyzed the expression of the downstream targets Axin2 and Cyclin D1. Only OE33 showed significant increased levels of Axin2 expression levels compared to controls. All other cell lines investigated did not show any alterations of Axin2 expression after Wnt3a treatment (Fig. 5b).

Furthermore, decreased expression levels of Cyclin D1 were found in the squamous (EPC-1, EPC-2), metaplastic (CP-A) and dysplastic (CP-B) cell lines after stimulation with Wnt3a. The cell line OE33 expressed increased levels of CyclinD1, while OE19 responded marginally (Fig. 5c). Westernblot analysis of phosphorylated β-catenin (Ser552), GSK3β (Ser9) and Akt (Ser473) revealed only marginal alterations after Wnt3a treatment (Fig. 5d-f). Only OE33 responded with an increased phosphorylation of β-catenin (Fig. 5e). We observed no phosphorylation of Akt in the metaplastic (CP-A) and dysplastic (CP-B) cell lines (Fig. 5f and Additional file 1: Figure S1).

### Expression of Wnt-signaling molecules in human specimens

**Fig. 6:**
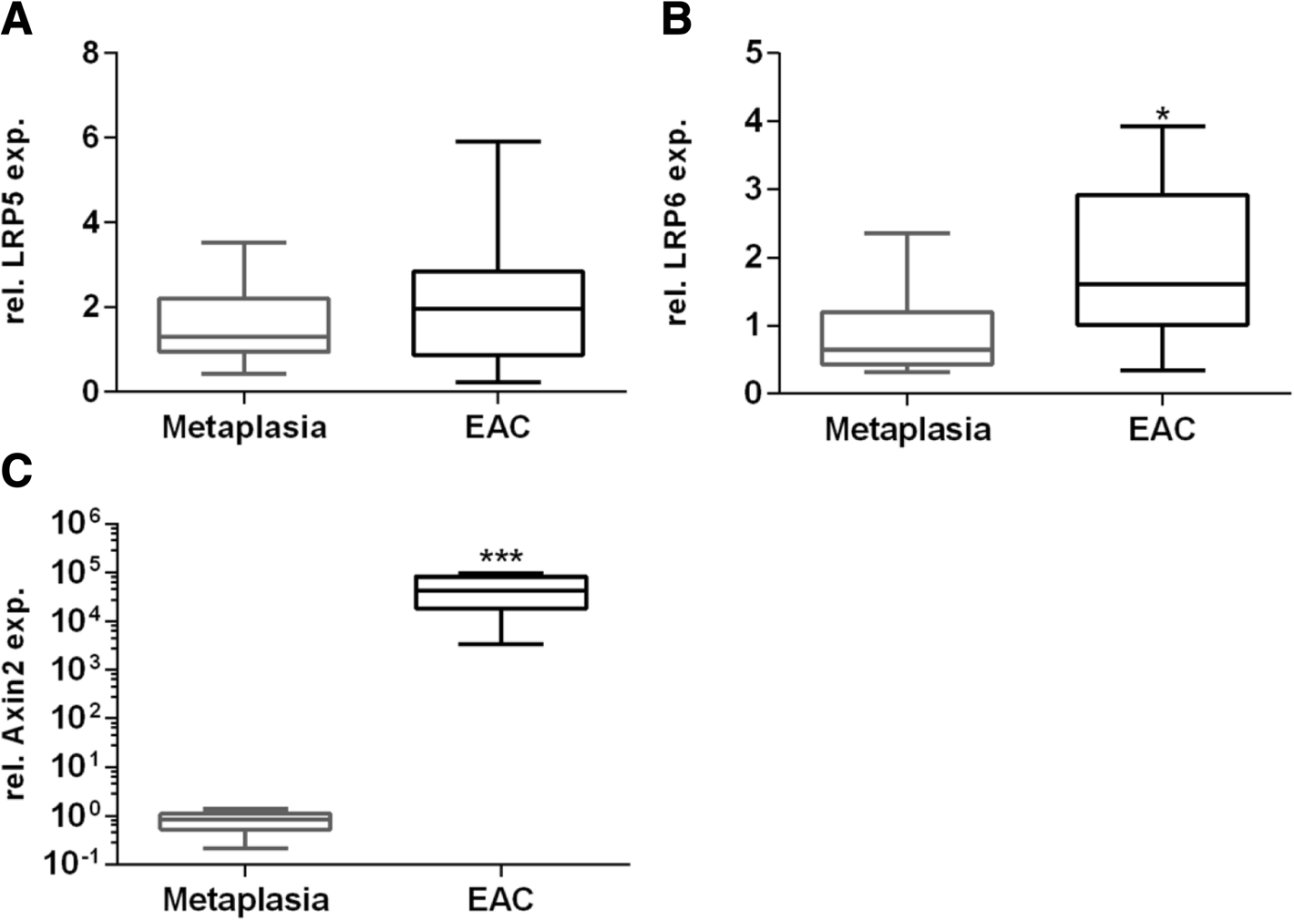
Expression of the Downstream target Axin2 and the co-receptors LRP5 in paraffin embedded biopsies. Expression of the co-receptors LRP5 (**a**) and LRP6 (**b)** and the Downstream target Axin2 (**c**) was analyzed in paraffin embedded biopsies by quantitative Real time RT PCR. Biopsies were cut in 3 μm sections and RNA was isolated. 125 ng of RNA were synthesized. Elevated LRP5 expression levels were found in EAC compared to metaplasia (**a**). Biopsies from EAC expressed significantly higher levels of LRP6 (**b**). In contrast to a marginal Axin2 expression in metaplasia, enormous levels of Axin2 were detected in EAC (**c**). Normalization was done with β-Actin. Values are shown as mean ± S.E.M. (Mann-Whitney test, * - p < 0.05, *** - *p* < 0.001)

Expression levels of LRP5/6 and Axin2 were analyzed in patient-derived biopsies. In concordance with results presented above, we found elevated expression levels of LRP5/6 in EAC as compared to metaplasia specimens. Additionally, the expression of Axin2 was significantly higher in EAC specimens as well (Fig. 6).

### WNT3A in immunohistochemistry

**Fig. 7:**
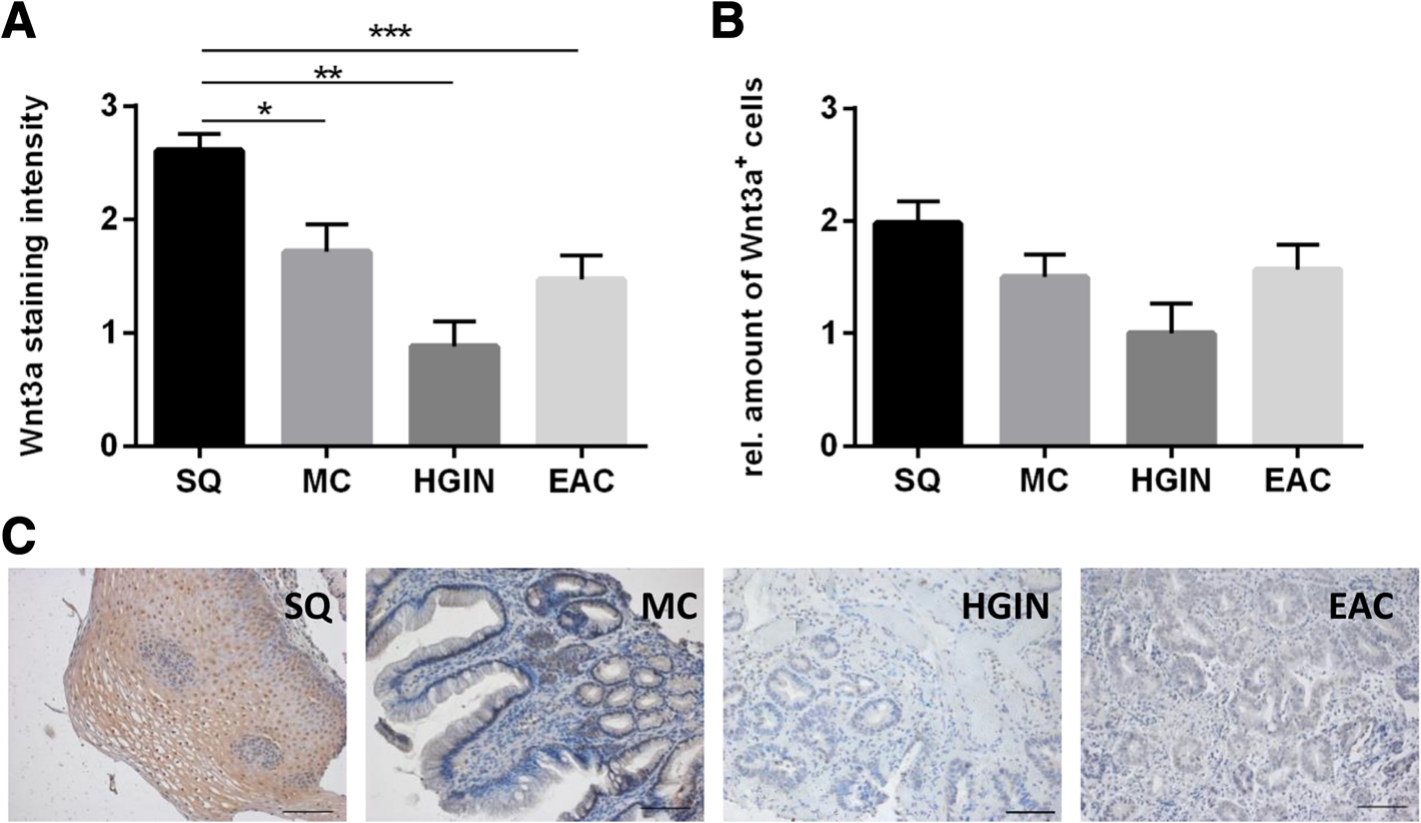
Immunohistochemically staining of WNT3A in metaplasia, HGIN and EAC. Immunohistochemical staining of WNT3A was performed and the staining index of WNT3A-positive cells (**a**) of squamous epithelial (SQ), metaplastic (MC), high-grade intraepithelial neoplasia (HGIN), and esophageal adenocarcinoma (EAC) cells in human specimens (**b**,**c**) were analyzed. The staining index used, describes staining as 1 < 30%, 2 < 60%, 3 < 100%. WNT3A protein localization, showed a higher staining index for SQ than MC, HGIN and EAC. Values are shown as mean ± S.E.M. (Kruskal-Wallis with Dunn’s correction, * - *p* < 0.05, *** - *p* < 0.001, n_SQ_ = 30, n_MC_ = 15, n_HGIN_ = 7, n_EAC_ = 15, bar-20 μm)

Immunohistochemical staining in Barrett’s metaplasia, HGIN, and adenocarcinoma of embedded specimens for WNT3A revealed a significant higher staining intensity of WNT3A positive cells in squamous epithelium (SQ) compared to intestinal metaplasia, HGIN and adenocarcinoma cells (Fig. 7a). The amount of WNT3A positive epithelial cells was slightly higher compared to metaplastic, HGIN, and adenocarcinoma cells (Fig. 7b/c). WNT3A was localized predominantly in the cytoplasm.

## Discussion

In this work, we provide an in-depth characterization of the Wnt-signaling pathway components on a cellular level using both, an in vitro cell culture model of Barrett’s esophagus and evidence from human data to elucidate changes in receptor, ligand, intracellular signaling cascade and downstream target of the Wnt-signaling pathway. Aberrations in Wnt-signaling had been observed in many malignancies, including colon cancer [11], hepatocellular carcinomas [20], non-small cell lung cancer [21], and esophageal adenocarcinoma [15–17, 22].

Characterizing Wnt-signaling pathway components in an in vitro Barrett’s esophagus cell culture model was carried out in squamous epithelium (EPC-1 and EPC-2), metaplastic (CP-A), high-grade dysplastic (CP-B) and in adenocarcinoma (OE33 and OE19) cells. We could show a wide spread of frizzled receptor expression throughout Barrett’s sequence, demonstrating mostly an increase in frizzled receptor expression in the more advanced Barrett’s cell lines for Fzd1, Fzd2, Fzd3, Fzd4, Fzd5, and Fzd7 (Fig. 2). While Fzd6 and Fzd8 did not show significant changes in their expression pattern, Fzd9 and Fzd10 were decreased. The ligand Wnt3a was silenced, beginning with the metaplastic cell line CP-A (Fig. 1a). However, Wnt5a was expressed in the squamous epithelial cells EPC-1 and EPC2, as well as, in the dysplastic cell line CP-B, while it was silenced in metaplastic cells (CP-A) and the two investigated EAC cell lines OE33 and OE19 (Fig. 1b). As frizzled receptor expressions are complex, the Wnt-ligands can mostly bind to one or more receptors. Therefore, in part, they are able to compensate for each other [23]. To increase intricacy even more, Wnt-signaling is dependent on co-receptors, such as LRP5 and LRP6, which hybridize the appropriate frizzled receptor after Wnt-ligand binding. While in vitro only, the LRP5 expression was significantly increased, we could show a significant higher expression of LRP6 in EAC compared to metaplasia specimens (Figs. 4 and 6). In vitro binding assays revealed Wnt3a binding to Fzd5 at a nanomolar level, which was increased in EAC cells (Fig. 2e) [23, 24]. The high binding affinity of Wnt3a was also confirmed by an in silico algorithm on a structural basis, where Wnt3a was predicted to bind Fzd1–3, Fzd5, and Fzd7–9 with high affinity and Wnt5a was found to bind Fzd3, Fzd5, Fzd8, and Fzd9 [25]. In general, Fzd5 has been described as a non-specific binder to almost all Wnt-ligands, except Wnt9a [25].

Since the Wnt-signaling pathway has been reported to be activated in the progression of Barrett’s esophagus, a robust characterization of its receptors is still missing [15, 17, 26]. The intracellular Wnt-signaling is mediated via GSK3β phosphorylation, which leads to a cytoplasmic accumulation of β-catenin [27]. In Barrett’s neoplastic progression, however, such an accumulation could not be found in metaplastic specimens [15]. In non-treated steady-state conditions, we observed no difference in the basal activation of GSK3β (Fig. 4d), whereas phosphorylation of β-catenin at Ser552 was decreased in the more advanced cell lines CP-B, OE33 and OE19 (Fig. 4c). Therefore, a Wnt-independent mechanism is discussed, which might be enhanced by the Akt/PI3K-pathway [28]. Since we could only show a robust Wnt3a expression pattern in cell lines, derived from normal squamous epithelium, EPC-1 and EPC-2 (Fig. 1a), as well as a weaker WNT3A staining in metaplastic, HGIN and esophageal adenocarcinoma specimens (Fig. 7), we were able to demonstrate a silencing of Wnt3a with the progression of the Barrett’s esophagus in vitro (cell lines) and in vivo (immunohistochemistry). However, biopsies from Barrett’s mucosa (4 of 7), HGIN (1 of 7) and EAC (2 of 7) showed Wnt3a expression, which might be from cells, others than Barrett’s epithelium, because the analyzed expression levels from biopsies contains several cell types, e.g. fibroblasts, immune cells, vascular cells, and others (Fig. 3). Another limitations of the study presented here are the relative small numbers of specimens investigated. Comorbidities, e.g. obesity, the duration of disease and pharmacologic kinetics of proton pump inhibitors might have an impact on the Wnt-signaling pathway, which needs to be investigated more in detail. The immunohistochemical evaluation carries out a higher accuracy to determine Wnt3a presence in vivo. Nevertheless, the Wnt-antagonizing factor Dkk1 is overexpressed in the more advanced Barrett’s stages, showing the highest abundance in EAC in both, in vitro (OE33 cells) and in vivo as well as activated β-catenin is also present in Barrett’s mucosa specimens [16, 19]. Both findings, the increase in Dkk1 and the decrease in Wnt3a support Wnt-independent β-catenin activation in Barrett’s esophagus. However, Wnt3a induced a 2fold increase in the Wnt-signaling pathway target Axin2 in OE33 cells, while Axin2 is increased in its basal expression levels with Barrett’s sequence (Fig. 5a/b and 6c). Wnt3a was not able to alter the phosphorylation status of GSK3β or β-catenin itself in any of the investigated cell lines (Fig. 5 )d/e. Surprisingly, the phosphorylation of Akt was diminished in the metaplastic (CP-A) and the dysplastic (CP-B) cell lines (Fig. 5f). Epigenetic remodeling in EAC and Barrett’s esophagus has already been reported, with silencing of the Wnt inhibitory factor-1 (WIF-1) and secreted frizzled receptors [29, 30]. However, loss-of-function or epigenetic alterations have not been described for β-catenin or APC, so far [31].

A potential alternative pathway, which might be involved in the Wnt-signaling pathway activation along the progression of Barrett’s esophagus, is the NF-κB-pathway, triggered by an increased TNF-α-receptivity in the more advanced stages of Barrett’s esophagus [32–34]. Acid-dependent and inflammation-induced damage leads to progression of Barrett’s esophagus, which could potentially contribute to TNF-α-promoted tumor growth and metastasis via PI3K/Akt, GSK3β, and NF-κB [35, 36].

We demonstrated for the first time a comprehensive analysis of all 10 frizzled receptors and their co-receptors LRP5 and LRP6 in an in vitro cell culture model of Barrett’s esophagus and in primary human specimens from normal squamous epithelium, Barrett’s mucosa, HGIN, and EAC.

## Conclusion

The in depth characterization of the Wnt-receptor and Wnt-ligand expression pattern will allow new insights into the activation of the Wnt-signaling pathway in Barrett’s esophagus and Barrett’s carcinoma, as well as in the pathomechanisms involved in the progression of Barrett’s esophagus. A concluding answer of the exact molecular level of Wnt-axis activation needs further elucidation and an exact molecular profile of both, a useful in vitro cell culture model as well as in vivo analyses of Barrett’s patients’ specimens.

## Additional file

Additional file 1: **Figure S1.** Phosphoryation of Akt, GSK3β and β-catenin after Wnt3a treatment in EPC1, EPC2, CP-A, CP-B, OE33 and OE19 cells. Representative Westernblots related to the analysis shown in Fig. 5. (PPTX 10686 kb)

## Abbreviations

Akt: Protein kinase B
APC: Adenomatous polyposis coli
BM: Barrett’s mucosa
BSA: Bovine serum albumin
cDNA: Complementary deoxyribonucleic acid
Dkk1: Dickkopf-related protein 1
Dsh: Dishevelled
EAC: Esophageal adenocarcinoma
FAP: Familiar adenomatous polyposis
FBS: Fetal bovine serum
FFPE: Formalin-fixed paraffin-embedded
Fzd: Frizzled
GERD: Gastroesophageal reflux disease
GSK3β: Glycogen synthase kinase 3 beta
HCC: Hepatocellular carcinoma
HGIN: High-grade intraepithelial neoplasia
hTERT: Human telomerase reverse transcriptase
LRP: Low-density lipoprotein receptor-related protein
NF-κB: Nuclear factor kappa-light-chain-enhancer of activated B cells
PBS: Phosphate-buffered saline
PCR: Polymerase chain reaction
PI3K: Phosphatidylinositol 3-kinase
rhWnt3a: Recombinant human Wnt3a
RIPA: Radioimmunoprecipitation assay buffer
RNA: Ribonucleic acid
RT: Reverse transcriptase
SDS: Sodium dodecyl sulphate
SFRP: Secreted frizzled receptor protein
SQ: Squamous epithelium
WIF-1: Wnt inhibitory factor-1
