# Supplementary material for: In-depth characterization of the Wnt-signaling/β-catenin pathway in an in vitro model of Barrett’s sequence
Source: BMC Gastroenterol. 2019 Mar 6;19:38. doi: 10.1186/s12876-019-0957-5 (PMC6404335; doi:10.1186/s12876-019-0957-5)
Supplement: Supplementary file 1 — Figure S1. Phosphoryation of Akt, GSK3β and β-catenin after Wnt3a treatment in EPC1, EPC2, CP-A, CP-B, OE33 and OE19 cells. Representative Westernblots related to the analysis shown in Fig. 5. (PPTX 10686 kb) [file 12876_2019_957_MOESM1_ESM.pptx]

## Slide 1
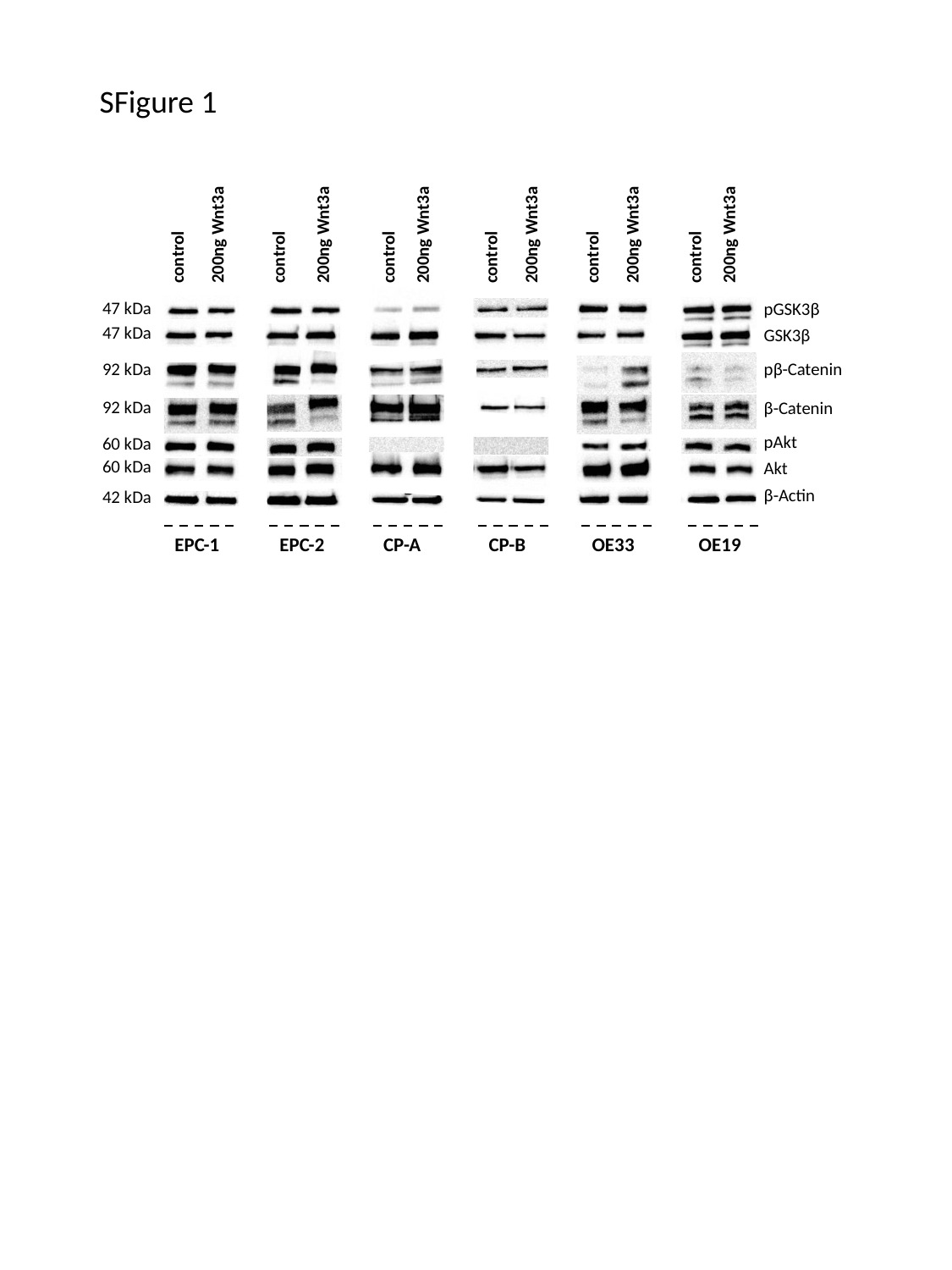

SFigure 1
200ng Wnt3a
200ng Wnt3a
200ng Wnt3a
200ng Wnt3a
200ng Wnt3a
200ng Wnt3a
control
control
control
control
control
control
47 kDa
pGSK3β
47 kDa
GSK3β
pβ-Catenin
92 kDa
92 kDa
β-Catenin
pAkt
60 kDa
60 kDa
Akt
β-Actin
42 kDa
EPC-1
EPC-2
CP-A
CP-B
OE33
OE19
